# Finding Value in Wastewaters from the Cork Industry: Carbon Dots Synthesis and Fluorescence for Hemeprotein Detection

**DOI:** 10.3390/molecules25102320

**Published:** 2020-05-15

**Authors:** Marta R. Alexandre, Alexandra I. Costa, Mário N. Berberan-Santos, José V. Prata

**Affiliations:** 1 Departamento de Engenharia Química, Instituto Superior de Engenharia de Lisboa, Instituto Politécnico de Lisboa, R. Conselheiro Emídio Navarro, 1, 1959-007 Lisboa, Portugal; malexandre@hovione.com (M.R.A.); acosta@deq.isel.ipl.pt (A.I.C.); 2 Centro de Química-Vila Real, Universidade de Trás-os-Montes e Alto Douro, 5000-801 Vila Real, Portugal; 3 Institute for Bioengineering and Biosciences, Instituto Superior Técnico, Universidade de Lisboa, Av. Rovisco Pais, 1049-001 Lisboa, Portugal; berberan@tecnico.ulisboa.pt

**Keywords:** cork, wastewater, carbon dots, fluorescence, sensor, haemoglobin, myoglobin, cytochrome *c*

## Abstract

Valorisation of industrial low-value waste residues was preconized. Hence, carbon dots (C-dots) were synthesized from wastewaters of the cork industry—an abundant and affordable, but environmentally-problematic industrial effluent. The carbon nanomaterials were structurally and morphologically characterised, and their photophysical properties were analysed by an ensemble of spectroscopy techniques. Afterwards, they were successfully applied as highly-sensitive fluorescence probes for the direct detection of haemproteins. Haemoglobin, cytochrome *c* and myoglobin were selected as specific targets owing to their relevant roles in living organisms, wherein their deficiencies or surpluses are associated with several medical conditions. For all of them, remarkable responses were achieved, allowing their detection at nanomolar levels. Steady-state and time-resolved fluorescence, ground-state UV–Vis absorption and electronic circular dichroism techniques were used to investigate the probable mechanisms behind the fluorescence turn-off of C-dots. Extensive experimental evidence points to a static quenching mechanism. Likewise, resonance energy transfer and collisional quenching have been discarded as excited-state deactivating mechanisms. It was additionally found that an oxidative, photoinduced electron transfer occurs for cytochrome *c*, the most electron-deficient protein. Besides, C-dots prepared from citric acid/ethylenediamine were comparatively assayed for protein detection and the differences between the two types of nanomaterials highlighted.

## 1. Introduction

In recent years, carbon dots (C-dots) gained increased visibility and interest in a variety of fields which include fluorescent bio-imaging and nanomedicine [1,2,3], sensory analysis [4,5] and photocatalysis [6,7], owing to their luminescent properties, good photostability, biocompatibility and low cytotoxicity [8,9].

The synthesis of C-dots encompasses both top-down and bottom-up approaches [8]. C-dots produced by bottom-up methods usually include solvothermal processes and/or chemical oxidation procedures, where organic compounds/polymers typically possessing acid, alcohol and amine functionalities are used as precursors [10].

Within aforementioned processes, production of carbon nanomaterials from industrial low-valued waste materials, for example, from several forest and vegetable sources and biomass wastes [11,12], is highly desirable and particularly useful, since it overall contributes to an effective circular economy of chemicals through recycling. Synthesis of luminescent C-dots produced from green precursors (e.g., fruits, fruit juices and peels, coffee grounds, vegetables and plant leaves) through benign and sustainable approaches have been pursued intensively in the last decade [12]. Waste paper [13] and waste frying oil [14] are two examples that have been used for production of C-dots with quantum yields ranging from 4–10%.

Particularly appealing sources for large scale production of C-dots are those based on abundant and affordable industrial aqueous effluents which represent, nonetheless, serious environmental issues that still needed to be solved. Very recently it has been revealed that highly fluorescent C-dots could be obtained from olive mill wastewaters following simple and environmental-friendly hydrothermal routes [15].

Cork industry wastewater (CIWW) is another abundant and recalcitrant effluent that results from cork industry activities. After harvesting, and a period of stabilization or rest, cork planks are subject to an operation called cooking for textural and plastic improvement of cork properties. Cork planks are cooked in nearly boiling water for a certain period of time (usually 1 h), or alternatively, using steam. The process water is reused for 20–30 extraction cycles. The resulting wastewater (ca. 200–400 L of CIWW for each tonne of processed raw cork), is rich in water-soluble extractives and suspended matter. Known components in spent water are phenol and polyphenol-based compounds (phenolic acids, flavonoids and hydrosoluble tannins); mono and neutral oligosaccharides; and acidic heteropolysaccharides (pectins) of the galacturonic acid family [16,17] and oxidation products thereof, overall exhibiting very low biodegradability parameters (BOD/COD = 0.16–0.21) [18]. To put the above figures into perspective, and taking only the amount of processed cork from the world’s largest cork producer (Portugal; 100,000 tonnes/year; 49.6% world quota) [19], huge amounts of CIWW need to be carefully and specifically treated each year. In spite of the efforts to treat and reuse this effluent using single or multidisciplinary treatment approaches [18,20,21,22,23,24,25,26,27,28], such processes seem to be yet far from being fully implemented in the cork industry.

Alternative approaches to tackle this challenging effluent, particularly those envisioning its valorisation through sustainable processes, are yet to be established. Within this framework, a process for the direct production of carbon-based nanomaterials from CIWW was recently disclosed [29]. Potential applications of this kind of C-dots are now under active scrutiny. The present study focuses on their ability to act as fluorescence probes for haemproteins.

The advantages of sensing schemes based on fluorescent C-dots, and the main principles underlying their sensitivity, have been recently reviewed [5]. In what regards the proteins’ detection and quantification, accurate, fast and cheap analytical methods are required for several analytical and research purposes, including medical diagnosis. Haem-containing proteins, such as haemoglobin, myoglobin and cytochrome *c*, play significant and vital roles in living organisms, whose abnormal amounts may lead to severe diseases. Therefore, great efforts toward their sensitive and selective detection are continuously being sought. After the first sparse reports on the use of C-dots for direct detection of haemoglobin [30,31,32], an increasing number of studies have appeared in the last two years having haemoglobin [15,33,34,35,36,37,38,39,40,41], cytochrome *c* [42,43] or myoglobin [44] as specific targets.

Herein, the synthesis and characterisation of C-dots isolated from cork wastewaters are described, along with the investigation of their sensory abilities toward relevant haemproteins via direct turn-off fluorescence-based assays. As will be shown, all three metalloproteins under evaluation, haemoglobin, myoglobin and cytochrome *c*, can be detected at nanomolar levels. A higher response was found for haemoglobin. The relevant mechanisms responsible for the observed reduction of C-dots luminescence are thoroughly discussed based on collected data from steady-state and time-resolved fluorescence, ground-state UV–Vis absorption and electronic circular dichroism techniques. Possible implications of the distinct structural nature of C-dots on the detection efficiency of proteins were also investigated, making use of C-dots prepared from citric acid/ethylenediamine precursors.

## 2. Results and Discussion

### 2.1. Characterisation of C-Dots

Analysis of characteristic functional groups at C-dots surface was performed by FTIR spectroscopy (Figure S1, Supplementary Materials). The most prominent bands appear at 3405 (O-H, str), 3270 (N-H, str), 2962 (CH_3_, str asym), 2935 (CH_2_, str asym), 2878 (CH_3_, str sym), 2855 (sh, CH_2_, str sym), 1654 (C=O, str, acid/amide I, C=N, quinones), 1592 (C=O, str, carboxylate, amide II, conjugated C=C, N-H, ben, amide II), 1385 (Ar-OH, str, C-O, str carboxylate, CH_3_ ben), 1120, 1080, and 1050 (C-O, str, OH, ben) cm^−1^. ^1^H-NMR spectra indicate the presence of aliphatic C-H resonances (0.8–2.7 ppm), a large component of C(O)-H/C(N)-H signals (3.2–4.5 ppm) and dispersed low intensity resonances corresponding to aromatic protons and carboxylic acids (6.3–9.0) over a broad aromatic envelope (Figure S2). The elemental analysis retrieved for C-dots after correction for ashes (23.9%) was as follows: C, 49.68%; H, 5.36%; N, 8.95%; O (calculated), 36.01%; and S < 0.3%. When comparing the CHN contents of CIWW and C-dots, a huge raise was observed for the N/C ratio (from 0.041 to 0.154), and a slight increase in H/C ratio (0.91 to 1.29) was registered. The nanomaterials obtained from citric acid (CA) and ethylenediamine (ED) (C-dots/CA) presented an elemental analysis of C, 54.24%; H, 5.75%; N, 20.67%; O (calculated), 19.34%; and S < 0.3%. A much higher N/C ratio (0.327) and a similar H/C ratio (1.260) were obtained in this case in comparison to C-dots, indicating a higher oxygen content in C-dots isolated from CIWW; for those prepared from CA, a more nitrogen-rich content was found. The FTIR of C-dots/CA (Figure S3) also points to that. Its ^1^H-NMR (Figure S4) spectrum reveals a single resonance due to aliphatic C-H (2.25 ppm), a set of C(O)-H and C(N)-H peaks (3.5–4.4 ppm) and well-resolved aromatic protons in the range 5.8–6.2 ppm, consistent with the presence of a molecular structure resembling that of citrazinic acid derivatives [45].

**Figure 1:**
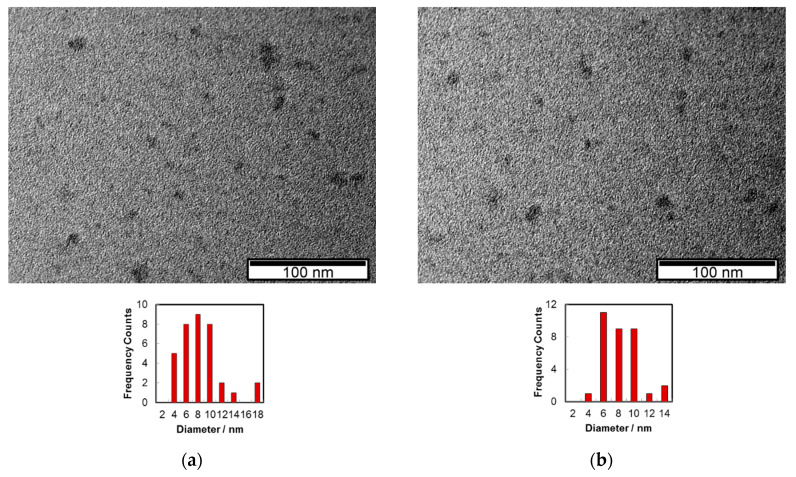
TEM images of C-dots, prepared from CIWW at 200 °C during 8 h, from two selected regions (**a**,**b**).

The morphology of C-dots was characterised by transmission electron microscopy (TEM) analysis. The nanomaterial images (Figure 1a,b) were captured after solvent evaporation of an aqueous solution of C-dots on the Cu grids/C film. The C-dots exhibit an average diameter around 8 nm.

A high dispersion of the apparent lateral sizes of C-dots, ranging from <5 nm to near 70 nm, may also be observed in some micrographs (Figure S5). It was found that a higher proportion of the large dark spots (>50 nm) tended to appear as the concentration of the aqueous solution used in the deposition increased. Thus, it seems conceivable that, as a result of strong aggregation of nanoparticles on the hydrophobic grid surface during the deposition process due to the very polar nature of surface groups present in the carbon nanoparticles, formation of large clusters occurs [46].

On the other hand, TEM images of C-dots/CA hardly revealed the presence of observable nanoparticles whatsoever (Figure S6). Indeed, a recent report [47] has pointed out that when hydrothermal temperatures in the range 150–200 °C are used in the synthesis of CA/ED-derived C-dots, polymer-like structures are mostly obtained.

### 2.2. Photophysical Properties

**Figure 2:**
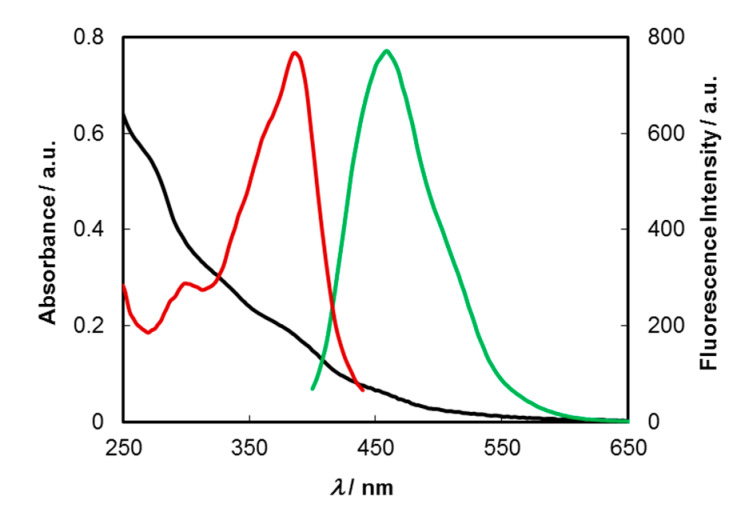
UV–Vis (black line), excitation (red line; monitored at 460 nm) and emission (green line; *λ*_exc_ = 380 nm) spectra of an aqueous solution of C-dots (0.1 mg/mL).

The absorption, excitation and fluorescence spectra of aqueous solutions (0.1 mg/mL) of C-dots are depicted in Figure 2.

**Figure 3:**
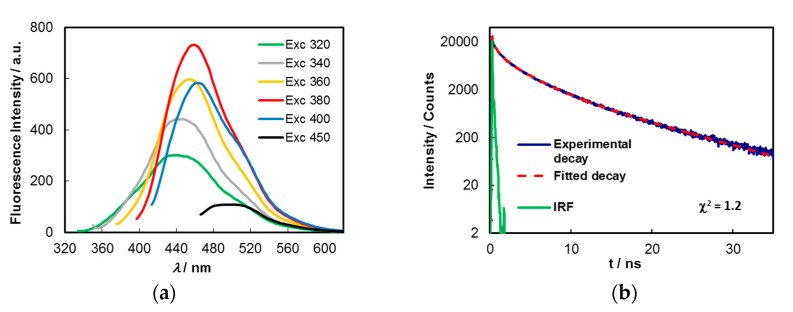
(**a**) Dependency of fluorescence emission of aqueous solutions of C-dots (0.1 mg/mL) upon excitation at different excitation wavelengths; (**b**) time-resolved intensity decay of a buffered solution (pH = 7.2) of C-dots obtained by the single-photon timing method under excitation at 340 nm.

The UV–Vis spectrum comprehends bands peaking at around 275, 325, 385 and 457 nm, and a vanishing absorption edge toward higher wavelengths, with the former assignable to π–π* transitions of carbon core (C_sp2_) and those at lower energies to n–π* and π–π* mixed transitions of carbonyl, carboxyl and imine functions at the surface and/or at inner sites of the carbon matrix. The excitation spectrum shows that the main chromophores responsible for the emission appear at around 345, 360 and 385 nm. A strong blue emission peaking around 460 nm is observed when aqueous solutions of C-dots are excited at 380 nm. The fluorescence quantum yields (*Φ*_F_), measured for aqueous solutions of C-dots synthesized from several batches, varied between 8.6% and 10.2% (*λ*_exc_ = 380 nm). This contrasts with that of pristine wastewaters for which a *Φ*_F_ = 0.52% (*λ*_exc_ = 360 nm) was found. C-dots display variable emission maxima, which are dependent on excitation wavelength. As shown in Figure 3a, significant redshifts are observed in the fluorescence spectra when the *λ*_exc_ is varied from 320 to 450 nm, while the emission intensities are progressively increased up to near 380 nm and then decrease, reflecting the profile of the excitation spectrum (Figure 2).

Time-resolved intensity decays of C-dots in an aqueous solution and a phosphate buffered solution (pH = 7.2) were obtained by the single-photon timing method with laser excitation and microchannel plate detection under 340 nm excitation (Figure 3b). It was found that the lifetime decays were best fitted by a sum of three exponentials, either in aqueous (*τ*_1_ = 9.08 ns (56.6%), *τ*_2_ = 2.51 ns (36.7%) and *τ*_3_ = 0.28 ns (6.8%)), or buffered solutions (*τ*_1_ = 9.38 ns (48.6%), *τ*_2_ = 3.12 ns (43.1%) and *τ*_3_ = 0.60 ns (8.3%)), yielding, respectively, average lifetimes (*τ*_ave_) of 6.1 and 6.0 ns.

The fractional contributions (f_i_) of each decay time to the emission intensities observed in steady-state measurements were calculated from the expression f_i_ = α_i_ × *τ*_i_/∑_j_ α_j_ × *τ*_j_ [48] (pp. 141–142), where α_i_/α_j_ are the pre-exponential factors. For the present system in aqueous solution, the three decay times translate into f_1_ = 84.5%, f_2_ = 15.1% and f_3_ = 0.31%. Deconvolution of the emission steady-state spectrum of C-dots in the same medium and excitation wavelength (*λ*_exc_ = 340 nm), is well-fitted (R^2^ = 0.9993) by three gaussian curves (Figure S7), which roughly represent the partial contributions of three different types of emissive entities to the overall emission. The relative contributions obtained from the areas under each curve were 82.5%, 16.6% and 0.9%. These figures strongly correlate with the fractional contributions retrieved above from time-resolved fluorescence data.

Multiexponential photoluminescence decays are a common feature of C-dots displaying excitation wavelength-dependent emission, indicating in general the presence of different emissive entities within the nanomaterials’ structure [8,10], with average lifetimes of 5 ns for emissions around 450 nm [10].

For C-dots/CA, the emission maximum at ca. 447 nm is independent of the excitation wavelength when this is varied between 320–380 nm (Figure S8). Such behaviour is recognized as being due to the presence of molecular fluorophores (citrazinic acid derivatives such as 5-oxo-1,2,3,5-tetrahydroimidazo[1,2-*a*]pyridine-7-carboxylic acid, the so-called IPCA) which are largely responsible for the observed optical properties of the bulk C-dots prepared by this route; namely, their high photoluminescence quantum yields, particularly when synthesized at temperatures below 200 °C [45,47,49]. Aqueous solutions of C-dots/CA display a *Φ*_F_ of 66.7% (*λ*_exc_ = 360 nm) and an *τ*_ave_ of 15.3 ns (χ^2^ = 1.0) (Figure S9), mainly composed by a long decay time component (*τ*_1_ = 15.54 ns (97.8%)) and a minor faster component (*τ*_2_ = 4.05 ns (2.2%)). The essentially single exponential decay is indicative of the existence of a sole particular type of molecular fluorophore residing in similar chemical environments within the nanoparticles. This is consistent with the excitation-independent emission behaviour that has been observed. The presence of IPCA, or similar derivatives, as dominating emission centres in these nanomaterials is substantiated by the photoluminescence lifetime of IPCA itself, which is around 14 ns [45].

### 2.3. Detection of Hemeproteins

The effects on the fluorescence emission of C-dots upon contact with selected haemproteins were first evaluated for haemoglobin. Haemoglobin (~64.7 kDa; human) is a tetrameric iron-containing protein responsible for the oxygen transport in mammals from respiratory organs to the rest of the body; its deficiency may lead to several well-known diseases, such as anaemia and leukaemia, whereas an overproduction of red blood cells leads to high Hgb levels (polycythaemia).

The spectrofluorimetric titrations were conducted in phosphate buffer (pH = 7.2) with haemoglobin in an oxidised state of haem iron (Fe(III), metHgb), as checked by the presence of characteristic bands of this form peaking at 500, 540, 576 and 632 nm (Figure S10) [50].

It was previously verified that the emission intensities of aqueous solutions of C-dots scale linearly with the concentration up to ca. 0.03 mg/mL (*λ*_exc_ = 380 nm) (Figure S11), showing no signs of self-quenching due to the aggregation phenomena of the nanoparticles. In addition, it was assured prior to fluorimetric experiments that the C-dots are photostable under the experimental conditions used. To this end, an air-equilibrated aqueous solution of C-dots (0.1 mg/mL) was continuously irradiated at 380 nm under the same conditions used in the sensing assays (pulsed xenon discharge lamp with an average power of 7.3 W at 50 Hz) for 5h, showing afterwards that the sample did not suffer any noticeable photodegradation or photobleaching (Figure S12; relative standard deviation (RSD) = 1.003%).

**Figure 4:**
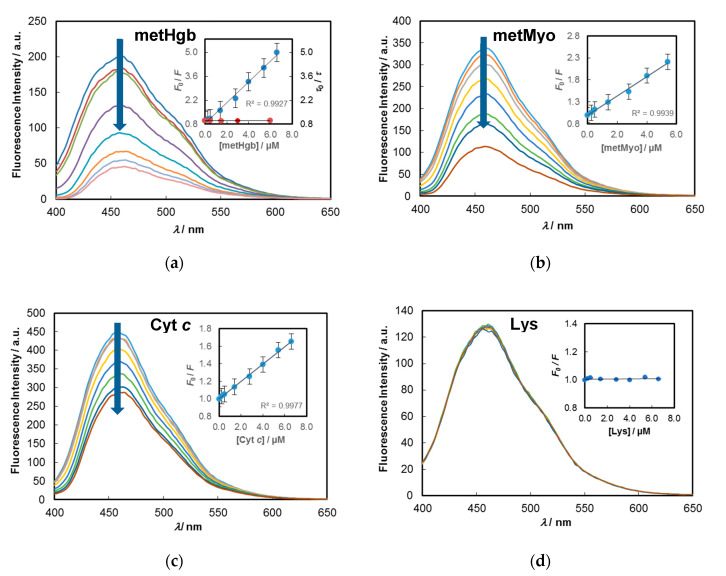
Emission spectra of C-dots (0.2 mg/mL) upon varying the amount of added protein (0, 0.25, 0.5, 1.4, 2.8, 4.0, 5.4, 6.6 µM) in phosphate buffer solution at 25 °C (pH = 7.2). Inset (**a**): Stern–Volmer plots obtained from steady-state (blue dots; *F*_0_/*F*; *λ*_exc_ = 380 nm) and time-resolved (red dots; *τ*_0_/*τ*; *λ*_exc_ = 340 nm) fluorescence data. Insets (**b**–**d**): Stern–Volmer plots from steady-state data (*λ*_exc_ = 380 nm). Error bars indicate the standard error.

Titrations were carried out at 25 °C by preparing a set of working solutions containing the C-dots (0.02 mg/mL) and metHgb concentrations in the range 0.25–6.6 µM. A high fluorescence quenching was noted (Figure 4) which reaches 80% reduction of initial emission upon addition of 6.6 µM of metHgb.

Integrated fluorescence emission spectra were used to quantify the relation between the decrease of emission of C-dots and the amount of added metHgb through the Stern–Volmer equation (see Materials and Methods). From the data (see inset in Figure 4a), a *K*_SV_ of 6.1 × 10^5^ M^−1^ was retrieved. This result clearly points to a high affinity between metHgb and the C-dots obtained from cork wastewater, allowing the detection and quantification of metHgb at very low levels. The limit of detection (LOD) was estimated as 7.8 nM and the limit of quantification (LOQ) as 26.0 nM. The sensorial efficiency toward haemoglobin, as measured by the Stern–Volmer constant, is higher or similar to other reported results [15,31,32,33,34,37].

For benchmarking purposes, the highly luminescent C-dots/CA prepared from citric acid/ethylenediamine were used as fluorescent probes for metHgb. Following the same titrimetric procedure, *K*_SV_ = 3.5 × 10^5^ M^−1^ (R^2^ = 0.996) was obtained (Figure S13). This value is 1.7-fold lower than that obtained for C-dots from CIWW, suggesting that the different structural nature of C-dots may play relevant roles in the establishment of such quite distinct sensitivities.

The response of C-dots to myoglobin in its oxidised form (metMyo), as judged from UV–Vis spectrum with Q bands appearing at 503 and 635 nm (Figure S14) [51], was next evaluated. Myoglobin (~17.7 kDa; from horse heart) contains a single peptide chain, and its main primary physiological role is to maintain intracellular oxygen storage and supply in skeletal muscle tissues and heart cells of vertebrates; the reduction of its availability by several diseases deeply affect muscle and cardiac functions. The observed quenching effects are displayed in Figure 4b. The experiment revealed that the quenching response to metMyo is 2.7-times lower than that observed for metHgb, with a *K*_SV_ of 2.2 × 10^5^ M^−1^ (LOD = 21.5 nM and LOQ = 71.5 nM). Following the same trend as that observed for metHgb, the response of C-dots/CA to metMyo (Figure S15) is also much lower (*K*_SV_ of 1.3 × 10^5^ M^−1^; R^2^ = 0.997).

Cytochrome *c* (Cyt *c*; ~12.4 kDa; from horse heart) is also a single-domain globular metalloprotein that works as an electron carrier in the mitochondrial respiratory systems of living organisms and in apoptosis; abnormal amounts of this protein have been shown to be correlated, for example, with several types of cancer and myocardial diseases. The data obtained from the spectrofluorimetric titration of C-dots with Cyt *c* in its ferric state is shown in Figure 4c. In this form, the peak maximum in the Q bands region appears at 532 nm (Figure S16) [52]. For this pair, an even lower response (*K*_SV_ of 1.0 × 10^5^ M^−1^; R^2^ = 0.998; LOD = 47.6 nm and LOQ = 158.6 nM) was obtained. Contrary to the former tested proteins, the quenching efficiency obtained with C-dots/CA was similar (*K*_SV_ = 1.1 × 10^5^ M^−1^; R^2^ = 0.989) (Figure S17).

The sensitivity exhibited by the cork-derived C-dots toward haemproteins was further checked against lysozyme (Lys; ~14.3 kDa; from chicken egg white), a highly basic protein devoid of a prosthetic centre. The assay showed no measurable quenching activity of Lys for C-dots fluorescence, as depicted in Figure 4d (average *F*_0_/*F* = 1.01; RSD = 0.69%). This result unequivocally shows that the high quenching efficiencies observed for the metalloproteins metHgb, metMyo and Cyt *c*, are not the results of mere aggregations of C-dots induced by the proteins followed by concomitant fluorescence self-quenching. Such nonspecific fluorophore–protein interactions have been put forward to rationalise quenching behaviours of certain conjugated polymer electrolytes (polyphenyleneethynylene and polyphenylvinylene type) in the presence of proteins [53,54], which drastically hamper their application as direct fluorescence probes of haemproteins. If the same rational were to apply to the systems under study, one would expect a significant quenching for Lys (pI = 10.5–11, [55]) which is positively charged (surface charge = +7) at pH = 7.2, and for Cyt *c* (pI = 9.6, [56]) which has also a highly surfaced charge (+9), compared to metHgb (pI = 6.9–7.3, [57]) and metMyo (pI = 6.8–7.4, [56]), which both have a net neutral surface charge at the working pH.

The effect of overall charge of a protein on the quenching of C-dots emission was evaluated. Before performing titration experiments at variable pH with the target proteins, the pH-dependency of fluorescence intensities of C-dots was first analysed using an excitation wavelength of 380 nm. As depicted in Figure S18, C-dots are most emissive at pH around 4–5, but display a large and useful working range for fluorimetric assays between pH 2 and 10.

Two proteins, metHgb and Cyt *c*, were used in the assay. The extent of quenching was evaluated at pH 5.0, 7.2 and 9.1 using acetate, phosphate and glycine buffers, respectively. The results are gathered in Table 1.

While Cyt *c* barely responds to changes in pH, the tetrameric protein metHgb is linearly sensitive to those variations. The different magnitudes of *K*_SV_ observed for metHgb are likely related to the changes in the overall surface charge of the protein. Indeed, at pH 5 the protein is positively charged; at pH 7.2 it is neutral; and at pH 9.1 it is negatively charged. In the case of Cyt *c*, the overall charge of the protein remains positive (pI = 9.6) over the entire pH range. From the above, it can be inferred that the electrostatic interactions and H-bonding developed between C-dots and proteins should play a substantial part in the quenching activity. The pH-responsive quenching may also allow for discrimination between haemproteins with different pIs.

### 2.4. Understanding the Sensitivity of C-Dots toward Hemeproteins

The great magnitudes of the Stern–Volmer constants attained by C-dots for all the metalloproteins under study clearly suggest that a static quenching mechanism is likely operating. Indeed, collisional quenching can be readily discarded on the basis of the apparent bimolecular quenching constant (*k*_q_^app^ = *K*_SV_/*τ*) retrieved from the data. For example, given the results for C-dots/metHgb pair (*K*_SV_ = 6.1 × 10^5^ M^−1^; *τ*_ave_ = 6.0 ns), a *k*_q_^app^ of 1.0 × 10^14^ M^−1^ s^−1^ is obtained, which is four orders of magnitude over that permitted for diffusion-limited quenching in aqueous solutions (ca. 1.0 × 10^10^ M^−1^ s^−1^) [48] (pp. 277–330). Moreover, the excited-state lifetimes of C-dots in the presence of increasing amounts of metHgb (Figure 4a; Table S1) showed no decrease whatsoever in average lifetimes (*τ*_0_/*τ* = 1), again ruling out dynamic quenching as a major route to the observed results. The unchanged nature of fluorescence decays in time-resolved experiments also rules out resonance energy transfer (RET) as a possible mechanism for the decrease of C-dots emission by means of a non-radiative transfer from C-dots (donor) to the protein (acceptor), since in such a case, shorter lifetimes are to be expected because the process depopulates the excited-state donor. Besides, high quencher concentrations (mM range) are needed for RET between unlinked donors and acceptors in solution [48] (pp. 277–330), which is not the case here. Although the possibility of RET occurrence has been indicated in a series of studies [15,30,36,37], no flawless demonstration of such a non-radiative reduction of emission was revealed.

Static quenching either involve the formation of a ground-state non-fluorescent complex or the development of less-specific interactions between the C-dots and the protein through a sphere of effective quenching [58]. While the former yields a linear *F*_0_/*F* plot (*F*_0_/*F* = 1 + *K*_SV_ [Q]), with *K*_SV_ being the association (binding) constant of fluorophore-quencher complex, the latter shows an exponential relationship of *F*_0_/*F* with the quencher concentration (*F*_0_/*F* = exp (*V*_q_*N*_a_ [Q], where *V*_q_ is the volume of the sphere-of-action and *N*_a_ is the Avogadro constant), which is responsible for the appearance of upward curvatures in Stern–Volmer plots at high quencher concentration regimes. However, at low quencher concentrations, such as those used in this work, a linear *F*_0_/*F* relationship is likely obtained since exp (*V*_q_*N*_a_ [Q]) turns into ≈ 1 + *V*_q_*N*_a_ [Q], with *V*_q_*N*_a_ = *K*_SV_ [58]. Therefore, both models can be admitted to justify the observed results.

**Figure 5:**
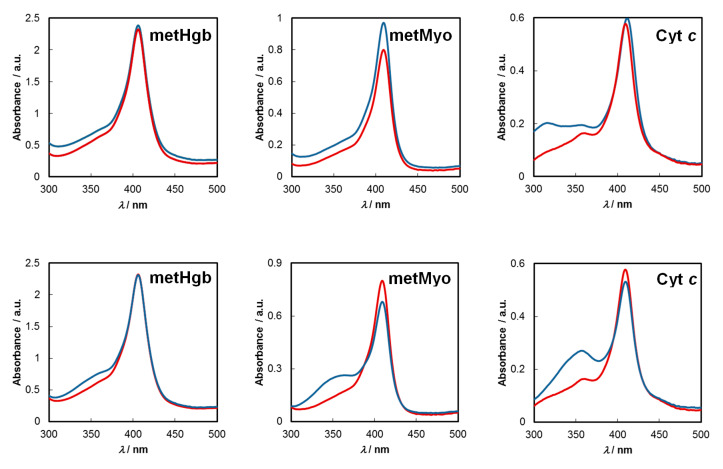
Ground-state absorption spectra for each protein (red line) and the corresponding C-dots–protein complex after subtraction of C-dots’ spectrum (blue line) for C-dots (upper panel) and C-dots/CA (lower panel) at pH = 7.2; [protein] = 6.6 µM.

Evidence for the formation of specific complexes may sometimes be obtained from analysis of ground-state UV–Vis spectra of the partners taking part in the sensing event. Figure 5 (upper panel) compares the absorption spectrum of each C-dots–protein pair after subtracting that of C-dots (C-dots-protein minus C-dots) with the absorption spectrum of the pristine proteins.

In all systems under study, perceptible changes occur in the UV–Vis region, indicating the formation of ground-state complexes. For all pairs, a hyperchromic shift was observed in the full extension of the wavelength range (300–500 nm), that occurring with C-dots-metMyo at the maximum of its Soret band (409 nm) being particularly notorious, accompanied by a slight broadening of the absorption band. The last feature proved common for metHgb and Cyt *c*. An additional redshift (~2 nm) was also observed at the absorption maximum (411 nm) of Cyt *c* after complexation with C-dots (see details on this point later on). Hyperchromicity [38] and hypochromicity [31,33] effects upon complexation have been noticed before.

When a similar study was undertaken for C-dots/CA, different outcomes resulted (Figure 5, lower panel). Thus, while for metHgb the hyperchromic effect was only seen before 390 nm, with no change observable at the absorption maximum of the protein (406 nm), for metMyo and Cyt *c* increases in absorption were similarly registered from 300 to 390 nm which were then followed by hypochromic shifts in the Soret band region maxima (409–410 nm).

The results from ground-state UV–Vis absorption show that the haemproteins bind to either type of C-dots, although in different degrees/types of association, reflecting their dissimilar surface and/or inner core structures, which likely influences the extent by which each C-dots’ fluorescence is turned off.

**Figure 6:**
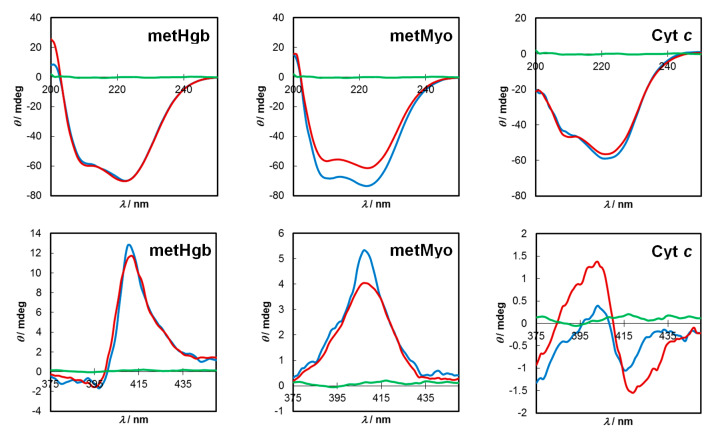
Circular dichroism spectra of proteins (red line), C-dots–protein complex (blue line) and C-dots (green line) in the far-UV (upper panel) and Soret (lower panel) regions at pH = 7.2.

Electronic circular dichroism (ECD) is a potent tool with which to study the secondary structures of proteins. For example, in the far-UV (below 250 nm) region, the typical ECD spectrum for α-helical structures shows a negative band at 222 nm (n–π* transitions of amides), and a negative band at around 208–209 nm coupled with a positive band at 190 nm due to exciton splitting of the π–π* transition of peptide bonds [59]. Furthermore, in the case of metalloproteins, their ECD spectra in the Soret region results from the coupled interactions of π–π* transitions of aromatic groups surrounding the haem and those of the porphyrin ring [59]. Any structural changes imparted by C-dots are expected to modify to some extent the spectra of native proteins in one or both regions. On the high energy side of the spectrum, H-bonding and electrostatic interactions may result from the carboxyl/carboxylate, amide and alcohol functionalities present in C-dots with the amino acid residues of the polypeptide chains leading to changes in the relative amounts of α-helices, β-sheets and random coil structures, whereas in the Soret region, hydrophobic interactions between conjugated C_sp2_ domains in the C-dot’s core and the haem pocket are likely to occur. Therefore, we investigate the influence of C-dots on the ECD spectra of proteins in the far-UV region (200–250 nm) and around the Soret bands (375–450 nm). Figure 6 (upper panel) shows the far-UV spectra of metHgb, metMyo and Cyt *c* in the absence and in the presence of C-dots (0.02 mg/mL) at pH =7.2.

The visual inspection of C-dots-metHgb/metHgb spectra revealed that no major changes occur in its secondary structure. Quantification of the fractions of secondary structures of the proteins were further accessed in the range 200–240 nm through K2D algorithm [60] as implemented in Dichroweb [61]. The amounts of α-helices, β-sheets and random coil structures in C-dots-metHgb ([metHgb] = 1.0 µM) were 39%, 8% and 53%, respectively, almost identical to those of native metHgb (41%, 6% and 53%), meaning that the initial protein conformation was conserved. This contrasts with reported works wherein the content of the α-helix dropped by 6% [38] or nearly 10% [31,37] upon interaction with C-dots. A different situation arises from the interaction of C-dots with metMyo ([metMyo] = 3 µM). For this pair, an increase in the α-helix content was observed (from 46% to 60%) with a reduction in the β-sheet component (23% to 7%). The secondary structure of Cyt *c* ([Cyt *c*] = 6.6 µM) in the presence of C-dots was not strongly altered; the calculated contents of α-helices, β-sheets and random coil structures were similar (31%, 11% and 58%, respectively).

The ECD spectrum of metHgb in the Soret region (Figure 6 (lower panel)) showed the typical positive Cotton effect peaking at 411 nm with a trough at 394 nm. Upon contact with C-dots, a slight enhancement in the positive dichroic signal occurred accompanied by a red shift (3 nm) in the negative peak. This reveals a specific interaction between the haem centre and C-dots. The conformational integrity of the haem is nevertheless preserved after complexation. The ECD of native Cyt *c* shows a negative couplet centred at around 410 nm. In the presence of C-dots the negative bisignate Cotton effect (peaking at 418 nm) decreases in intensity and blue shifts to 416 nm, whereas the positive counterpart decreases even more, yielding an asymmetric couplet. For metMyo, a significant increase in the intensity of the positive monosignate Cotton effect at 408 nm was observed. The ECD results retrieved from the Soret region demonstrate that noticeable interactions occur between the haem groups of the proteins and the C-dots, thereby reinforcing the idea of a static quenching mechanism.

Of course, the existence of a sphere of effective quenching cannot be excluded based on the above grounds, and it probably co-exists with the static mechanism. In this last scenario, and within the quenching sphere, a fast oxidative photoinduced electron transfer (PET) may occur between the excited-states of the electron-rich C-dots and the lowest unoccupied molecular orbitals (LUMO) of oxidised haem centres (Fe(III). This transient charge-transfer complex would return afterwards to the ground-state by a non-radiative process, thereby explaining the observed fluorescence quenching. An analogous mechanism was proposed for the quenching of poly[lithium 5-methoxy-2-(4-sulfobutoxy)-1,4-phenylenevinylene] by Cyt *c*, although in this case an additional strong aggregation-induced self-quenching also occurs [53].

**Figure 7:**
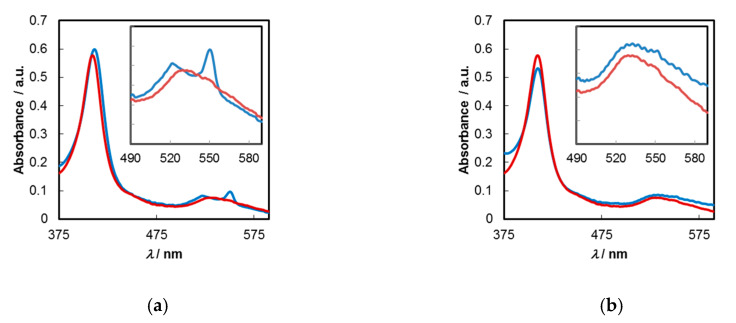
Absorption spectra of Cyt *c* (red line (**a**,**b**)) and Cyt *c* after contact with C-dots (blue line (**a**)) and C-dots/CA (blue line (**b**)); [Cyt *c*] = 6.6 µM, [C-dots] = 0.02 mg/mL and [C-dots/CA] = 0.005 mg/mL in buffered solutions at pH = 7.2.

For a pure PET mechanism, the greater the exergonicity between the excited electron donor (C-dots) band energy and the LUMO of the acceptor (Fe(III)-porphyrin on protein), the higher the observed quenching. Solely taking into consideration the reduction potentials of the three haemproteins (*E*^0^_metHgb_ = −70 mV; *E*^0^_metMyo_ = −236 mV; and *E*^0^_Cytc_ = +20 mV vs. SCE at 25 °C in aqueous solutions at pH = 7) [62], which translate into LUMO energies of −4.33 eV, −4.16 eV and −4.42 eV, respectively [63], the most favourable electron transfer should occur with the strongest oxidizing protein, Cyt *c*. Clear evidence for the existence of a contributing PET mechanism comes precisely from this protein. In its oxidised haem state, the UV–Vis spectrum of Cyt *c* shows two bands peaking at 409 and 532 nm (Figure 7a). After contact with C-dots, a reduction to Fe(II) state occurs, leading to the appearances of new bands at 411, 522 and 550 nm (Figure 7a) which are characteristic of the reduced form [52].

This undoubtedly demonstrates the occurrence of PET in this particular system. This fact should also be linked to the previously made observation on the changes of dichroic signals around the haem group. When the same study was performed with C-dots/CA, no noticeable changes occurred in the shape of the UV–Vis spectrum in the Soret and/or Q bands regions, suggesting the unfeasibility of electron transfer from these carbon materials (Figure 7b).

For the other two proteins, metHgb and metMyo, no perceptible changes assignable to metal reduction occur in the Q bands region.

Additionally, the ability of C-dots to participate in PET events involving unbound Fe(III) was investigated. Using aqueous solutions of Fe(III) in a concentration range of 10–68 µM (*λ*_exc_ = 380 nm, 25 °C), a *K*_sv_ of 3.9 × 10^3^ M^−1^ was retrieved (R^2^ = 0.994) (Figure S19a). When a similar experiment was conducted with ferrous ion, no measurable quenching activity was registered (Figure S19b; RSD = 1.44%). This unequivocally shows again that C-dots are able to behave as electron donors in PET mediated processes.

In our systems, as demonstrated above, static quenching, apparent static quenching (sphere of effective quenching) and oxidative photoinduced electron transfer mechanisms may all be contributing mechanisms to the observed fluorescence quenching of C-dots. In addition, possible inner filter effects (IFEs) should also be considered in order to get a more precise and wider picture of the observed emission reductions. As depicted in Figure S20, a spectral overlap of the absorption bands of the haemproteins with the excitation and emission bands of C-dots exist. This possibly leads to primary and/or secondary hetero IFEs. In the first case, a net reduction of incident light reaching the C-dots due to absorbing proteins may cause a decrease of their emission intensity. This trivial reduction in the emission of C-dots is linearly dependent on the molar extinction coefficient (*ε*) of the protein and its concentration, meaning that IFEs are more prone with the highest absorbing protein, metHgb. When the extinction coefficients of the three native proteins at 380 nm and pH = 7.2 were compared, ratios of *ε*_Hgb/_*ε*_Myo_ = 3.8 and *ε*_Myo_/*ε*_Cyt_ = 1.35 were found. These figures do not scale accordingly to those obtained for the corresponding *K*_sv_ ratios (*K*_sv-Hgb_/*K*_sv-Myo_ = 2.8 and *K*_sv-Myo_/*K*_sv-Cyt *c*_ = 2.2. Partial evidence for the existence of primary IFEs may be obtained by changing the excitation wavelength in the titration experiment. Thus, taking metHgb as an example, when the *λ*_exc_ was set to 340 nm, a wavelength where the molar attenuation coefficient of the protein was reduced by a factor of 1.9 compared to 380 nm, the resulting *K*_SV_ suffered only a 1.2-fold decrease. This lack of proportionality shows that primary hetero IFEs are not entirely responsible for the observed reduction on C-dots emission, or even perhaps do not play a major role on that matter. This point was further demonstrated by comparing the Stern–Volmer constants with the molar extinction coefficients of metHgb at various pH under 380 nm excitation. It was found that, albeit a reduction (15%) on the extinction coefficient occurs on going from pH = 7.2 (*ε*_Hgb_ = 1.39 × 10^5^ M^−1^ cm^−1^) to pH = 5.0 (*ε*_Hgb_ = 1.19 × 10^5^ M^−1^ cm^−1^), the corresponding *K*_SV_ saw an increase of 22% (see Table 1). Thus, IFEs cannot account for the increase in quenching efficiency at acidic pH. As argued before, such enhancement should be due to increased interactions between the positively charged protein surface and C-dots, which in turn raises the binding constant responsible for the static quenching.

For a molecular fluorophore whose emission maximum does not change with the excitation energy (Kasha rule), such a variation of *K*_SV_ at different excitation wavelengths could be safely connected to IFEs, and appropriate mathematical corrections of the emission intensity applied to get a more real value of *K*_SV_. Analysis of this issue in the present system is complicated by the fact that for C-dots, having wavelength-dependent emissions due to different chromogenic entities in their heterogeneous compositions, the nature (and location within the nanoparticle) of the excited chromophores at 380 nm is not necessarily the same as those excited at 340 nm, meaning that different interactions with proteins may result for the two populations of excited chromophores, which in turn may modulate *K*_SV_ at different extents, precluding a direct evaluation and correction of IFEs in these systems.

The radiative energy transfer leading to secondary hetero IFEs does not seem to occur to a great extent in our systems, since the shape of the emission bands of C-dots upon contacting with the proteins is not distorted nor is the emission maxima redshifted [58].

Previous accounts have traced IFEs as responsible for the observed quenching of C-dots’ fluorescence [15,32,35,40]. In some others works, although not recognised, strong IFEs should exist as indicated by the heavily distorted emission spectra in the region of spectral overlap [31,36,37].

## 3. Materials and Methods

### 3.1. Materials

The cork industry wastewater (CIWW) was collected from an industrial cork processing unit in Montijo, Portugal. After collection, the CIWW was kept refrigerated at −15 °C in polyethylene bottles. Before each use, the CIWW was thermally equilibrated at ca. 20 °C and homogenised through vigorous shaking.

Human haemoglobin (lyophilized powder; Sigma H7379, Sigma-Aldrich Corp., St. Louis, MO, USA; methaemoglobin form (metHgb)), cytochrome *c* from equine heart (≥95% (SDS-PAGE), Aldrich C2506, Sigma-Aldrich Corp., St. Louis, MO, USA; ferricytochrome *c* form (Cyt *c*)), myoglobin from equine heart (≥90% (SDS-PAGE), Aldrich M1882, Sigma-Aldrich Corp., St. Louis, MO, USA; metmyoglobin form (metMyo)), lysozyme from chicken egg white (Lys, ≥90%, Aldrich L6876, Sigma-Aldrich Corp., St. Louis, MO, USA), quinine hemisulphate monohydrate (QS, >98%, Fluka, Sigma-Aldrich Corp., St. Louis, MO, USA), citric acid monohydrate (CA, 99.8%, Fisher Scientific, Pittsburgh, PA, USA), ethylenediamine (ED, >99.5%, Fluka, Sigma-Aldrich Corp., St. Louis, MO, USA) and iron(II) chloride (98%, Aldrich, Sigma-Aldrich Corp., St. Louis, MO, USA) and iron(III) chloride.6H_2_O (97%, Merck KGaA, Darmstadt, Germany) were used as received. Aqueous buffered solutions at pH = 5.0, 7.2 and 9.1 were prepared by standard methods from acetate (50 mM), phosphate (50 mM) and 20 mM glycine (59 mM) buffers, respectively. Ultrapure water (Milli-Q, Millipore; Merck KGaA, Darmstadt, Germany) was used throughout the experiments.

### 3.2. Procedure for the Synthesis of C-Dots from Cork Industry Wastewater

C-dots were synthesized from CIWWs by a hydrothermal process, following a described protocol [29]. Briefly, ethylenediamine (ED; 44 µL, 0.66 mmol) was added to 20 mL of CIWW (total solids (TS) concentration around 6.3 g/L, pH = 5.5), and the mixture heated in a 100 mL capacity Teflon-lined high-pressure reactor (Parr model 4560) equipped with pressure, temperature and stirring sensors/controllers (Parr, model 4843), for 8h at 200 °C (autogenous pressure of 15 bar). After cooling to room temperature (ca. 25 °C), the contents were filtered through a 0.20 µm cellulose membrane. Acetone (100 mL) was then added to the dark brown filtrate and the mixture centrifuged (Hermle Labortechnik Z383K centrifuge) at 4500 rpm for 10 min. The light amber supernatant was evaporated until dryness. The residue was dried at 105 °C under vacuum yielding 60.1 mg of C-dots as a brown solid (36.6% *w*/*w* based on TS of CIWW and ED). The as-prepared solid nanomaterials were used directly in their characterisations and in protein fluorimetric assays upon dispersion in ultrapure water. Aqueous solutions of C-dots could also be directly obtained after the centrifugation step through smooth removal of acetone under vacuum at ca. 40 °C, yielding amber solutions of C-dots in a concentration around 3.0 mg/mL.

### 3.3. Procedure for the Synthesis of C-Dots from Citric Acid

The synthesis of C-dots from citric acid was adapted from a reported procedure [64]. In a typical experiment, 2.19 g (10.0 mmol) of citric acid (CA) monohydrate and ethylenediamine (ED; 1.0 mL, 15.0 mmol) were dissolved in deionized water (20 mL) and the mixture heated in a Teflon-lined high-pressure reactor (see above) for 4 h at 175 °C (autogenous pressure of 10 bar). The dark brown reaction mixture was filtered through a 0.20 µm cellulose membrane after cooling to room temperature. After addition of acetone (100 mL), a dark brown oil got deposited and the amber supernatant was decanted. After removal of solvents from the supernatant at 50 °C and vacuum drying at 105 °C, 1.0 g (32.5% *w*/*w* based on CA and ED) of a brown solid (C-dots/CA) was isolated. These materials were used as so in sensing assays upon dispersion in ultrapure water.

### 3.4. Instruments and Methods

FTIR spectra were acquired on a Bruker Vertex 70 (Bruker Optik GmbH, Ettlingen, Germany) as KBr pellets in transmission mode using a spectral resolution of 2 cm^−1^. Whenever possible, band assignments are tentatively indicated by the nature of the vibration (stretching (str), bending (ben), symmetric (sym) and asymmetric (asym)) corresponding to particular functional groups.

^1^H-NMR (400 MHz) spectra were collected on a Bruker AVANCE II+ spectrometer (400 MHz, Bruker BioSpin AG, Fällanden, Switzerland) at 25 °C using a concentrated solution/dispersion of C-dots in D_2_O. The reported chemical shifts (δ/ppm) are internally referenced to residual solvent signals (^1^H-NMR: D_2_O, 4.790 ppm).

Elemental analyses (CHNS) were determined on a Carlo Erba EA 1108 analyser (Carlo Erba, Milan, Italy) at C.A.C.T.I., Universidad de Vigo, Spain.

The transmission electron microscopy (TEM) was carried out on a Hitachi H-8100 microscope (lantanium hexaboride filament, 200 kV, Hitachi, Tokyo, Japan), with a point-to-point resolution of 2.7 Å using a 200 mesh copper grid covered with a Formvar Carbon film, at MicroLab, Universidade de Lisboa, Portugal. The aqueous solutions/dispersions of C-dots were deposited in the support and left to evaporate naturally at ca. 20 °C. The diameter of the particles was estimated by Image J (version 1.50i, National Institutes of Health, Bethesda, MD, USA) [65].

Ground-state UV–Vis spectra were recorded on a Jasco V-530 or a Jasco J-815 CD spectrometer (Jasco Inc., Tokyo, Japan) using 1-cm quartz cells. Steady-state fluorescence spectra were collected on a PerkinElmer LS45 fluorimeter (PerkinElmer, Waltham, MA, USA) using a 1-cm quartz cuvette with right angle (RA) geometry at 25 °C in air-equilibrated conditions. Electronic circular dichroism (ECD) and UV–Vis spectra were acquired simultaneously on a Jasco J-815 CD spectrometer using 1 cm quartz cells at 20 °C using a Jasco Peltier type accessory CDF-426S/426L (Jasco Inc., Tokyo, Japan) as temperature controller. Aqueous solutions or aqueous buffered solutions were used in the determinations.

Time-resolved fluorescence intensity decay data were obtained by the single-photon timing method. The light source was a mode locked DPSS Nd:YVO_4_ green laser (Vanguard 2000-HM532, Spectra Physics Inc., Santa Clara, CA, USA) synchronously pumping a cavity dumped dye laser (701, Coherent, Santa Clara, CA, USA, delivering frequency-doubled 3–4 ps pulses of about 40 nJ/pulse at 3.4 MHz) working with 4-(dicyanomethylene)-2-methyl-6-(4-dimethylaminostyryl)-4*H*-pyran (DCM). Emission light was detected by a Hamamatsu 2809U-01 microchannel plate photomultiplier (Hamamatsu Photonics, Hamamatsu, Japan). Excitation wavelength was 340 nm. Ensemble fluorescence decays data were analysed with a sum of exponentials.

The fluorescence quantum yields (*Φ*_F_) of C-dots in aqueous solutions were determined by the slope method [66] using quinine sulphate as reference standard (*Φ*_F_ = 0.54 in 0.1 M H_2_SO_4_) under RA geometry and 360 or 380 nm excitation in air equilibrated conditions. Inner filter effects during quantum yield measurements were prevented by keeping the optical density of the sample and reference below 0.05 at the excitation wavelength.

Sensing experiments were performed with aqueous solutions of C-dots (typically at 0.02 mg/mL) obtained under the conditions reported above. For titration experiments, the test solutions were prepared in aqueous buffered solutions (50 mM phosphate buffer, pH = 7.2, unless stated otherwise) at various C-dots: protein ratios by varying the concentration of proteins from 0.25 µM to up to 6.6 µM in separated volumetric flasks. The air-equilibrated solutions were kept at 25 °C for 1 min prior to spectrofluorimetric readings. After exciting at the desired wavelength (380 nm, unless stated otherwise), the areas under the fluorescence emission curves were taken to derive the sensitivity of the nanoparticles towards each protein making use of the Stern–Volmer equation *F*_0_/*F* = 1 + *K*_SV_ [Q], where *F*_0_ and *F* are the fluorescence intensities of C-dots in the absence and presence of the protein (quencher), [Q] is the quencher concentration and *K*_SV_ is the Stern–Volmer constant. The limit of detection (LOD) was calculated from the expression LOD = 3 σ/slope of the calibration curve, where σ is the standard deviation of the instrument response for 16 blank measurements of C-dots [0.02 mg/mL]; the limit of quantification (LOQ) was estimated from 3.3 LOD. A similar setup and data treatment was followed for the C-dots prepared from citric acid/ethylenediamine (C-dots/CA).

The pHs of solutions were determined at ca. 25 °C with a pH Denver Instrument 215 (Denver Instruments, Bohemia, NY, USA).

Estimation of secondary structures of proteins and their complexes with C-dots was performed with the Dichroweb analysis webserver [61,67,68,69] using the K2D algorithm (Kohonen neural network method) [60].

## 4. Conclusions

Carbon nanomaterials prepared from wastewaters of cork industry have the ability to function as efficient fluorescent probes for the direct detection of haemproteins. The tetrameric protein haemoglobin displayed the highest response, followed by myoglobin and cytochrome *c*, all of them in their oxidised states. The most probable mechanisms underpinning the observed luminescence reduction of C-dots were investigated through titration assays using steady-state and time-resolved fluorescence, ground-state UV–Vis absorption and electronic circular dichroism methods. Evidence from UV–Vis and ECD data indicates a predominant static quenching mechanism. Furthermore, the unchanged decay profiles and excited-state lifetimes of C-dots in the absence and in the presence of increasing amounts of metHgb, in addition to the high magnitude of Stern–Volmer constants reached for all the metalloproteins, strongly negates the possibility of collisional quenching and resonance energy transfer as main deactivating processes of C-dots’ excited-state energy. Noteworthy, a photoinduced electron transfer mechanism was found to additionally operate with the most powerful oxidizing protein, Cyt *c*, as revealed by the change in the oxidation state of the iron-haem. IFEs cannot solely account for the observed *K*_SV_, as shown from pH-dependent titration experiments.

At last, the importance of the structural features of C-dots to the detection efficiency for haemproteins was also highlighted by comparing two distinct types of C-dots from different origins.

## 5. Patents

Patent applications have been filed for part of the work described in this publication.

## Figures and Tables

**Table 1:** Sensory efficiencies of C-dots toward metHgb and Cyt *c* at various pHs.

pH	metHgb	Cyt *c*
	*K*_SV_/M^−1^ (R^2^)	*K*_SV_/M^−1^ (R^2^)
5.0	7.4 × 10^5^ (0.992)	1.0 × 10^5^ (0.991)
7.2	6.1 × 10^5^ (0.993)	1.0 × 10^5^ (0.998)
9.1	5.2 × 10^5^ (0.996)	1.0 × 10^5^ (0.977)

## Supplementary Materials

The following are available online, Figures S1–S20 and Table S1.
