# Supplementary material for: Finding Value in Wastewaters from the Cork Industry: Carbon Dots Synthesis and Fluorescence for Hemeprotein Detection
Source: Molecules. 2020 May 15;25(10):2320. doi: 10.3390/molecules25102320 (PMC7287723; doi:10.3390/molecules25102320)
Supplement: Supplementary file 1 [file molecules-25-02320-s001.pdf]

Finding Value in Wastewaters from the Cork Industry: Carbon Dots Synthesis and Fluorescence for Hemeprotein Detection

Marta R. Alexandre [1,†], Alexandra I. Costa [1,2], Mário N. Berberan-Santos [3] and José V. Prata [1,2,*]

[1] Departamento de Engenharia Química, Instituto Superior de Engenharia de Lisboa, Instituto Politécnico de Lisboa, R. Conselheiro Emídio Navarro, 1, 1959-007 Lisboa, Portugal; malexandre@hovione.com (M.R.A.); acosta@deq.isel.ipl.pt (A.I.C.)
[2] Centro de Química-Vila Real, Universidade de Trás-os-Montes e Alto Douro, 5000-801 Vila Real, Portugal
[3] Institute for Bioengineering and Biosciences, Instituto Superior Técnico, Universidade de Lisboa, Av. Rovisco Pais, 1049-001 Lisboa, Portugal; berberan@tecnico.ulisboa.pt
[†] Present address: Hovione FarmaCiencia SA, Sete Casas 2674-506 Loures, Portugal
[*] Correspondence: jvprata@deq.isel.ipl.pt; Tel.: +351-218317172

Supplementary Materials

Table of Contents Page No.

Figure S1. FTIR spectrum of C-dots ---------- II
Figure S2. ^{1}H-NMR spectrum of C-dots ---------- II
Figure S3. FTIR spectrum of C-dots/CA ---------- III
Figure S4. ^{1}H-NMR spectrum of C-dots/CA ---------- III
Figure S5. TEM images of C-dots prepared from CIWW ---------- IV
Figure S6. TEM images of C-dots/CA ---------- IV
Figure S7. Spectral deconvolution of emission spectrum of C-dots ---------- V
Figure S8. Dependency of fluorescence emission of C-dots/CA ---------- V
Figure S9. Time-resolved fluorescence decay of C-dots/CA ---------- V
Figure S10. Q bands of metHgb ---------- VI
Figure S11. Concentration effects on the emission of C-dots ---------- VI
Figure S12. Photodegradation of C-dots ---------- VI
Figure S13. Emission spectra of C-dots/CA upon addition of metHgb ---------- VII
Figure S14. Q bands of metMyo ---------- VII
Figure S15. Emission spectra of C-dots/CA upon addition of metMyo ---------- VII
Figure S16. Q bands of Cyt *c* ---------- VIII
Figure S17. Emission spectra of C-dots/CA upon addition of Cyt *c* ---------- VIII
Figure S18. Photoluminescence of aqueous solutions of C-dots at various pH ---------- VIII
Figure S19. Fluorimetric titration of C-dots with Fe(III) and Fe(II) ---------- IX
Figure S20. Emission and excitation spectra of C-dots superimposed with the absorption spectra of proteins ---------- IX
Table S1. Decay data of C-dots in the presence of metHgb ---------- IX

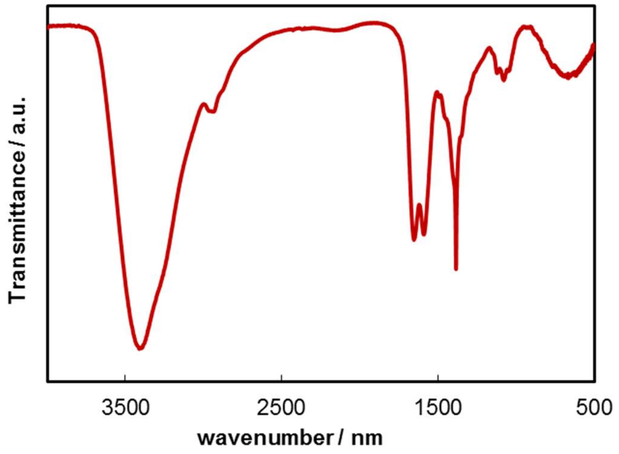

Figure S1. FTIR spectrum of C-dots.

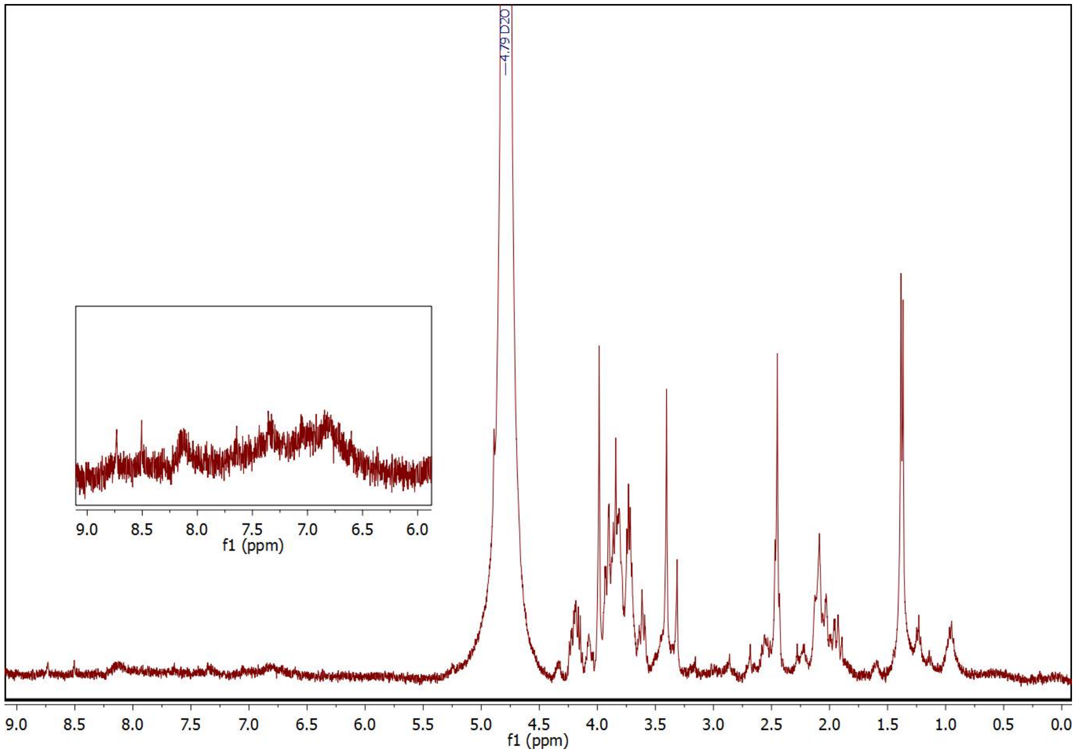

Figure S2. ^{1}H-NMR spectrum of C-dots (400 MHz, D_2O, 25 °C).

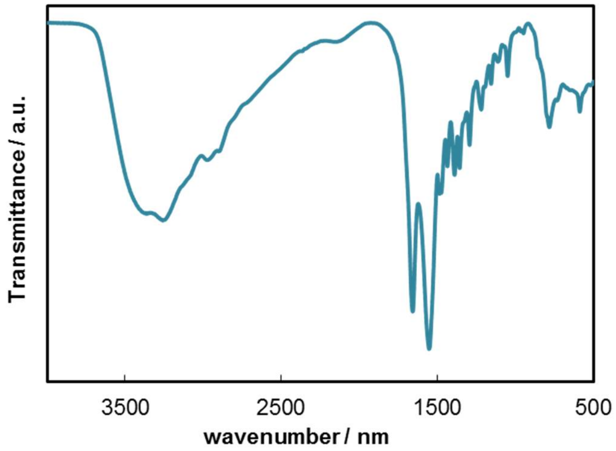

Figure S3. FTIR spectrum of C-dots/CA.

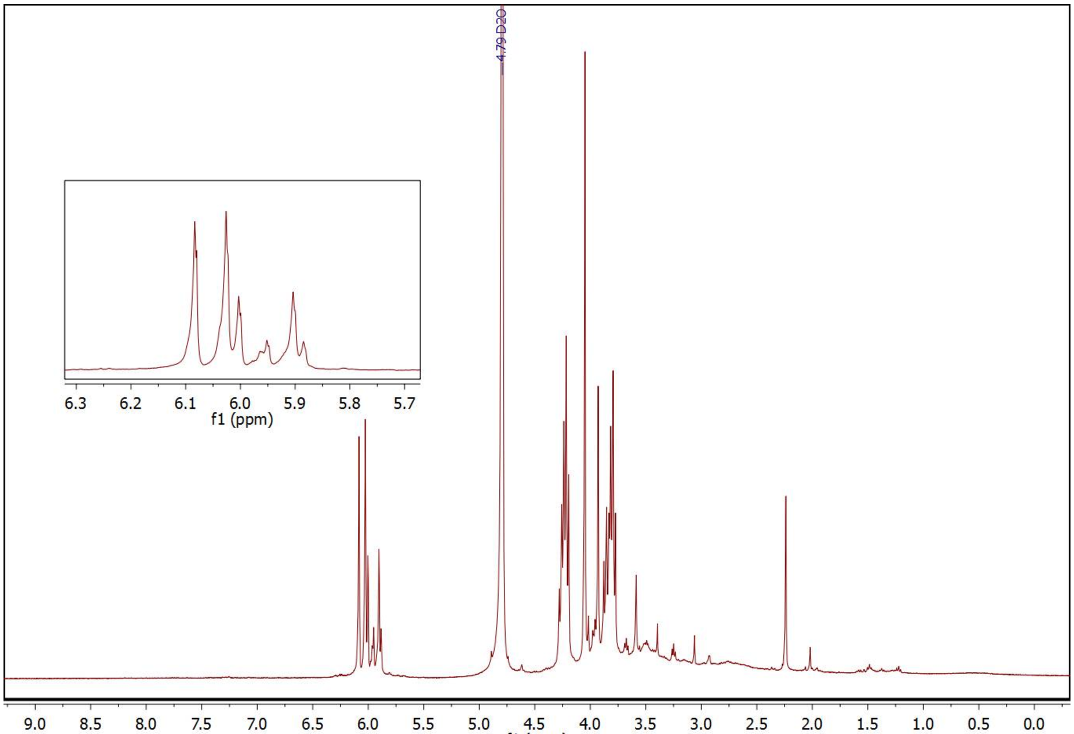

Figure S4. ^{1}H-NMR spectrum of C-dots/CA (400 MHz, D_2O, 25 °C).

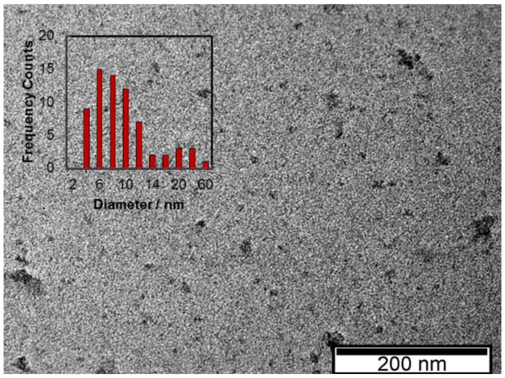

Figure S5. TEM images of C-dots prepared from CIWW at 200 °C during 8 h.

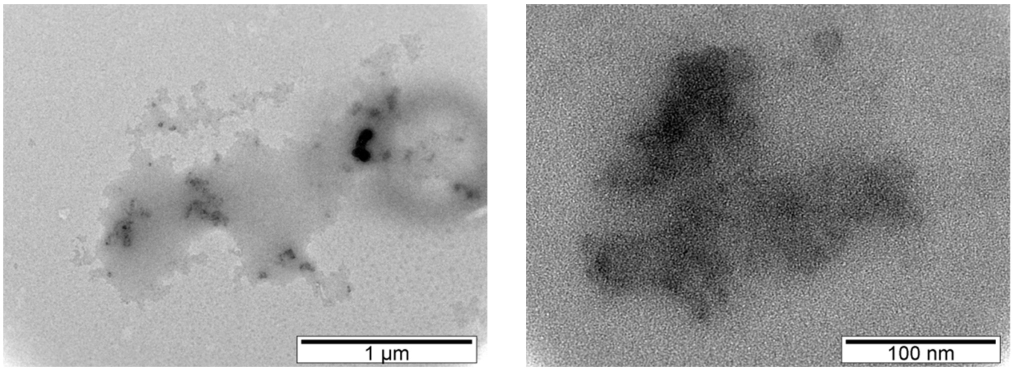

Figure S6. TEM images of C-dots/CA prepared from CA and ED at 175 °C for 4 h.

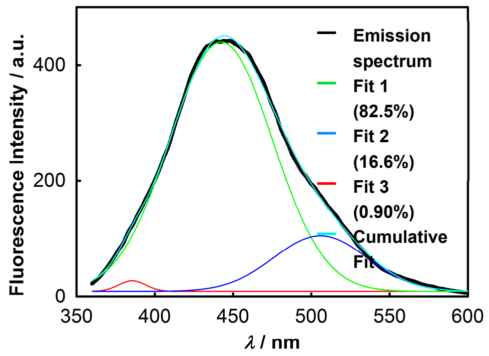

Figure S7. Spectral deconvolution of emission spectrum of C-dots (λ_{exc} = 340 nm).

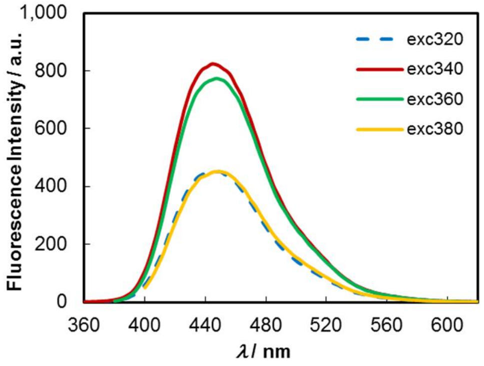

Figure S8. Dependence of fluorescence emission of aqueous solutions of C-dots/CA (0.005 mg/mL) on illumination at different excitation wavelengths.

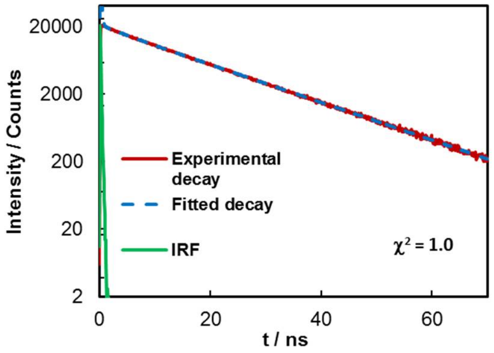

Figure S9. Time-resolved intensity decay of a buffered solution (pH = 7.2) of C-dots/CA obtained by the single-photon timing method under excitation at 340 nm.

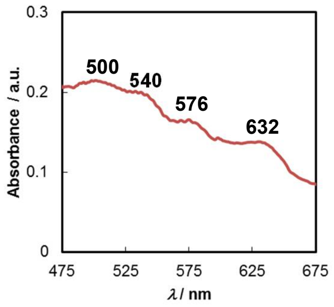

Figure S10. Q bands of metHgb (6.6 μM) in phosphate buffer solution (pH = 7.2) at 25 °C.

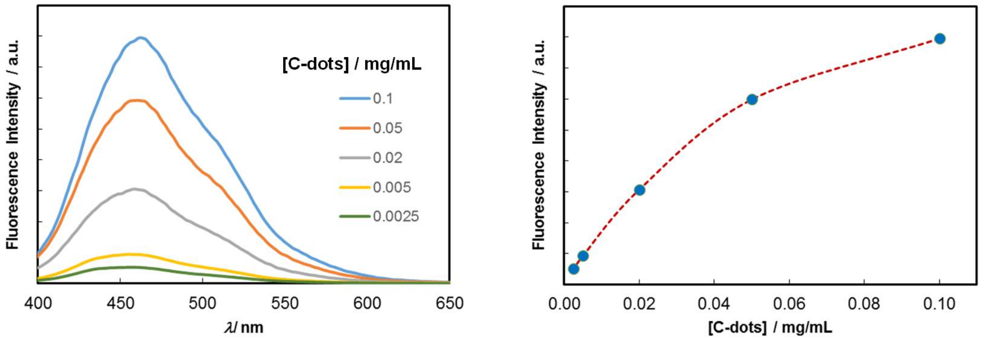

Figure S11. Concentration effects on the fluorescence intensity of aqueous solutions of C-dots (λ_{exc} = 380 nm).

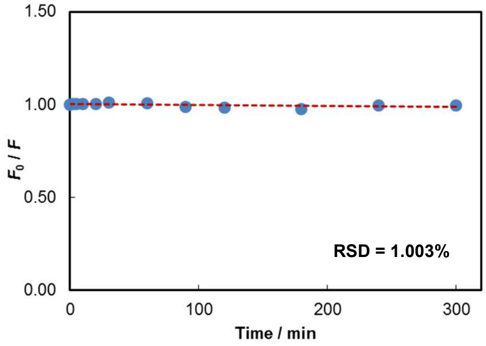

Figure S12. Change of fluorescence emission of C-dots (0.1 mg/mL) upon continuous irradiation at 380 nm for 5 h.

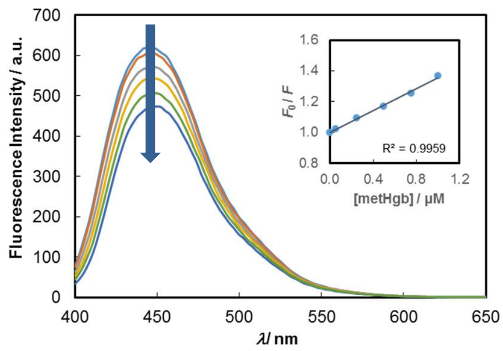

Figure S13. Emission spectra of C-dots/CA (0.005 mg/mL) upon addition of metHgb (0, 0.05, 0.25, 0.5, 0.75, 1.0 μM) in phosphate buffer solution at 25 °C (pH = 7.2). Inset: Stern-Volmer plot obtained from steady-state fluorescence data (λ_{exc} = 380 nm).

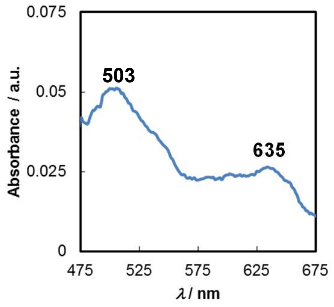

Figure S14. Q bands of metMyo (6.6 μM) in phosphate buffer solution (pH = 7.2) at 25 °C.

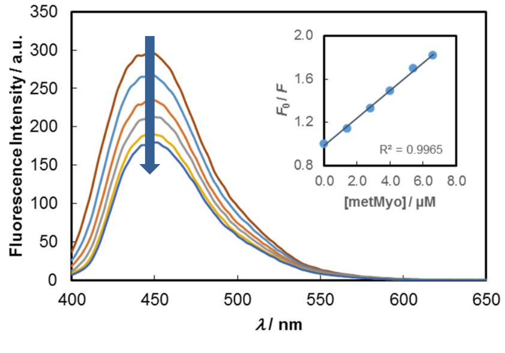

Figure S15. Emission spectra of C-dots/CA (0.005 mg/mL) upon addition of metMyo (0, 1.4, 2.8, 4.0, 5.4, 6.6 μM) in phosphate buffer solution at 25 °C (pH = 7.2). Inset: Stern-Volmer plot obtained from steady-state fluorescence data (λ_{exc} = 380 nm).

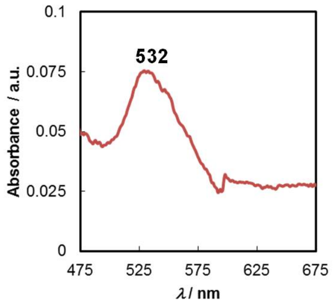

Figure S16. Q bands of Cyt *c* (6.6 μM) in phosphate buffer solution (pH = 7.2) at 25 °C.

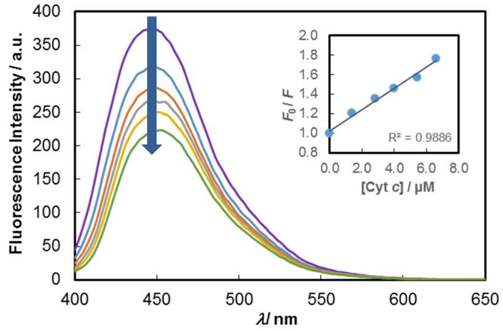

Figure S17. Emission spectra of C-dots/CA (0.005 mg/mL) upon addition of Cyt *c* (0, 1.4, 2.8, 4.0, 5.4, 6.6 μM) in phosphate buffer solution at 25 °C (pH = 7.2). Inset: Stern-Volmer plot obtained from steady-state fluorescence data (λ_{exc} = 380 nm).

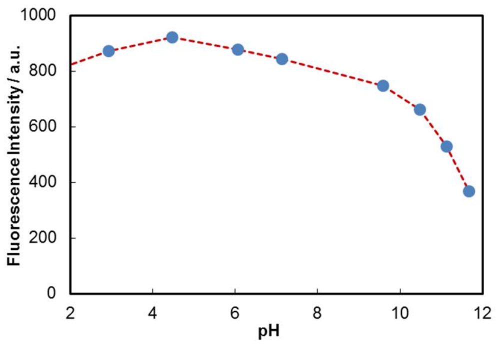

Figure S18. Photoluminescence of aqueous solutions of C-dots (0.1 mg/mL) at various pH; λ_{exc} = 380 nm.

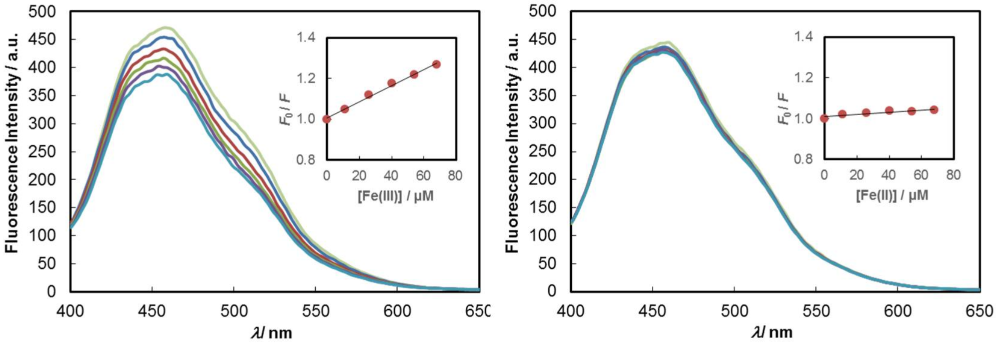

(**a**) (**b**)

Figure S19. Emission spectra of C-dots (0.005 mg/mL) upon varying the amount of added Fe(III) (**a**) and Fe(II) (**b**) in aqueous solution at 25 °C. Insets: Stern-Volmer plots obtained from steady-state fluorescence data (λ_{exc} = 380 nm).

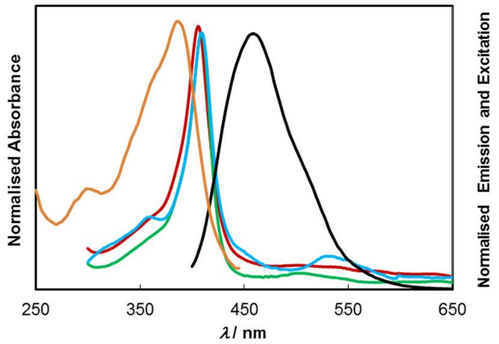

Figure S20. Emission (black line; λ_{exc} = 380 nm) and excitation (orange line; monitored at 460 nm) spectra of C-dots overlaid with the absorption spectra of metHgb (red line), metMyo (green line) and Cyt *c* (cyan line).

Table S1. Decay data of C-dots in the presence of metHgb.

[metHgb]/μM	τ_1/ns (%)	τ_2/ns (%)	τ_3/ns (%)	τ_{ave}/ns	χ^2	τ_0/τ
0	9.38 (48.6)	3.12 (43.1)	0.60 (8.3)	6.0	1.2	1.00
0.5	9.18 (50.8)	2.96 (41.8)	0.55 (7.4)	5.9	1.2	1.00
1.5	9.53 (47.5)	3.22 (43.2)	0.75 (9.3)	6.0	1.2	0.99
3	9.43 (47.7)	3.19 (42.8)	0.72 (9.5)	5.9	1.2	1.01
6	9.56 (45.6)	3.32 (43.9)	0.7 (10.5)	5.9	1.1	1.01
